# Managing Vibration Training Safety by Using Knee Flexion Angle and Rating Perceived Exertion

**DOI:** 10.3390/s21041158

**Published:** 2021-02-07

**Authors:** Long-Ren Chuang, Wen-Wen Yang, Po-Ling Chang, Vincent Chiun-Fan Chen, Chiang Liu, Tzyy-Yuang Shiang

**Affiliations:** 1 Department of Combat Sports and Chinese Martial Arts, Chinese Culture University, Taipei 11114, Taiwan; allen@ulive.pccu.edu.tw (L.-R.C.); blc77.tw@gmail.com (P.-L.C.); 2 Department of Sports Medicine, China Medical University, Taichung 406040, Taiwan; wwy@mail.cmu.edu.tw; 3 Engineering Program, Loyola University Chicago, Chicago, IL 60660, USA; cchen17@luc.edu; 4 Graduate Institute of Sports Equipment Technology, University of Taipei, Taipei 11153, Taiwan; cliu@utaipei.edu.tw; 5 Center for Sport Science and Technology, National Tsing Hua University, Hsinchu 300044, Taiwan; 6 Department of Athletic Performance, National Taiwan Normal University, Taipei 11677, Taiwan

**Keywords:** knee, RPE, vibration, exercise, subjective measurement

## Abstract

Whole-body vibration (WBV) is commonly applied in exercise and rehabilitation and its safety issues have been a major concern. Vibration measured using accelerometers can be used to further analyze the vibration transmissibility. Optimal bending angles and rating of perceived exertion (RPE) evaluations have not been sufficiently explored to mitigate the adverse effect. Therefore, the aims of this study were to investigate the effect of various knee flexion angles on the transmissibility to the head and knee, the RPE during WBV exposure, and the link between the transmissibility to the head and the RPE. Sixteen participants randomly performed static squats with knee flexion angles of 90, 110, 130, and 150 degrees on a WBV platform. Three accelerometers were fixed on the head, knee, and center of the vibration platform to provide data of platform-to-head and platform-to-knee transmissibilities. The results showed that the flexion angle of 110 degrees induced the lowest platform-to-head transmissibility and the lowest RPE (*p* < 0.01). A positive correlation between RPE and the platform-to-head transmissibility was observed. This study concluded that a knee flexion of about 110 degrees is most appropriate for reducing vibration transmissibility. The reported RPE could be used to reflect the vibration impact to the head.

## 1. Introduction

Whole-body vibration (WBV) has been studied in the fields of sports and rehabilitation to enhance training outcomes [1,2,3,4]. Over the last two decades, the use of WBV as a physical exercise and therapy has become a promising approach for improving the strength and muscle power of lower extremities [5,6,7,8]. It has been demonstrated in several studies that WBV improves the aging process of bone, cartilage, muscles, and tendons [8,9,10]. However, this has aroused the attention to potential injuries that may occur during vibration exposures and the ideal vibration stimulus magnitudes that one can tolerate and sustain. In fact, if a human body is exposed to vibration over a long period, it is possible to induce head-related (such as vertigo and visual impairment) [11,12,13,14] and joint-related musculoskeletal injuries [15,16]. Because of these negative consequences, in the domain of work sciences, the vibration magnitude and exposure time have been systematically standardized according to ISO 2631-1 [17]. By contrast, in the WBV training applied to athletes, the vibration magnitude is largely beyond the range of the ISO safety requirements [18]. Notwithstanding the potential injury and adverse consequences induced by vibration, WBV training is still being applied routinely to gain possible benefits from the approach. To strike a balance between training effectiveness and one’s well-being, the optimal design of WBV training protocols should be further investigated to provide better training guidelines.

The change of knee joint angle, being a critical factor in the WBV training protocol, not only affects the training stimulation of the knee extensor [19,20], but also modulates the vibration transmission for safety [18,21,22]. To determine the maximized capability of WBV training, most previous studies have investigated the effect of different knee angles (90–150 degrees) on the muscle activation of knee extensors [19,20,23]. These studies have discovered that by performing a squat with deep knee flexion, higher muscle activations of knee extensors can be induced, especially when compared with performing a squat with less knee flexion. Possible reasons of this phenomenon include the muscle activation to counteract increased external loads [22] and increased sensitivity of the muscle spindle to induce tonic reflex vibration due to increased muscle length [24]. On the other hand, for training safety, a few studies have investigated the effect of different knee angles on vibration transmission to the upper and lower segments by means of accelerometers, especially on the head and knee regions. They documented that both the standing position (180 degrees) and small knee flexion angles cause higher vibration transmission to the head and knee regions than a relatively low squat (90–140 degrees) [18,25,26]. It is usually mentioned that the additional damping from knee extensors, induced by WBV during squatting, may alter the vibration transmission from the knee to hip regions [24,27,28].

Studies have investigated the effect of different knee flexion angles on transmissibility to the head and knee, and the influences remain controversial. A previous study of Muir, Kiel, and Rubin [18] found slight attenuation of vibration transmission to the head but marketable attenuation of that to the knee at 90 degrees, compared with that at 135 degrees. In contrast, Tankisheva et al. [27] study reported no difference in the vibration transmissibility to the head and knee regions between the knees flexed at 110 and 135 degrees. Therefore, the comparisons of transmissibility among different flexion angles from half to partial squats (90–140 degrees) have not yet been well understood. Muscle activation and muscle stiffness mutually regulate vibration transmissibility. Muscle activation of knee extensors has been mentioned to mitigate vibration transmission [27]. High muscle stiffness (more rigid) produces a lower damping effect in response to perturbation, leading to a great magnitude of vibration transmission [29,30]. Although muscle activation increases from a partial to a half squat [19,23], concurrently, muscle stiffness becomes higher, thereby lowering the dissipating vibration energy. If these conditions are taken into account, the suitable knee flexion angle may not be at a knee flexion angle of 90 degrees, but probably between the knee flexion angle of 90 and 140 degrees. Moreover, long-term WBV training has been widely utilized to improve the physical and functional abilities of the elderly [31] and knee injury individuals [4]. The higher and accumulative vibrations could potentially cause acute and chronic injuries to the head and fragile joints [12,13,16,18]. Therefore, maintaining proper knee angles to diminish vibration transmission to the head and knee is important and desirable. 

For designing training protocols, an objective assessment of vibration exposure is needed to provide important information; in clinical practice, subjective evaluation is a potential approach for monitoring training programs. The subjective rating of perceived exertion (RPE) is considered an index to assess the training loads and intensity after WBV vibration due to a positive relation with muscle activation [32]. However, there is little evidence of the RPE for the assessment of safety. The human perception of vibration signals is a comprehensive analysis of multiple signals, including the level of vibration stimulation [32,33] and the sensation of body [33,34]. Accordingly, subjective RPE measurement may also provide useful information for practical application to evaluate the vibration impact on the body segments of each individual. The vibration of musculoskeletal structures can be an effective exercise intervention, but the intensity and safety of vibration treatment and training should be evaluated, not only objectively, but also from a personalized perspective to avoid any unwanted effects. Therefore, the purposes of this study were (1) to compare the effect of varying knee flexion angles (from 90 to 150 degrees with an increase of 20 degrees) on vibration transmissibility to the knee and head, the RPE during WBV exposure, and further to determine which knee bending angle is better for reducing the transmissibility to the knee or head; (2) to investigate the relationship between the RPE and the transmissibility to the head or knee. We hypothesized that the knee flexion angle would be a major factor of vibration transmissibility and RPE would be correlated with the vibration transmissibility to the head of the exercising subject. 

## 2. Materials and Methods

Sixteen male college students (age: 20.3 ± 1.1 years; height: 171.7 ± 5.3 cm; weight: 65.8 ± 7.9 kg) participated in this study and engaged in about three 60 min recreational activities per week, such as jogging and cycling. This study was approved by the Institutional Review Board of the local university. Written informed consent was given to each participant and was signed prior to the experiment. Each participant was informed of the details of the procedures and the experimental risks of motion sickness. During the experiments, if the participants felt uncomfortable or experienced dizziness or nausea, the experiments were terminated.

**Figure 1:**
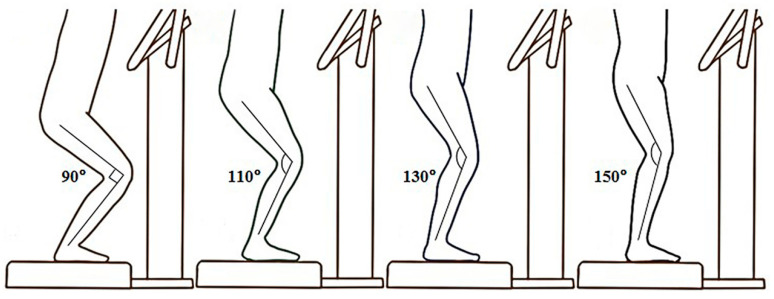
Postures of various knee angles under whole-body vibration stimulation.

Each participant squatted with four different knee flexed angles at 90, 110, 130, and 150 degrees (Figure 1) in a random and counterbalanced order on the vertical WBV platform (Zen Pro 6903, Tonic Fitness Technology Inc., Tainan, Taiwan). A goniometer was used to ensure the knee was placed in the right angular position. The receiving vibration stimulation was set at a frequency of 20 Hz and a peak-to-peak amplitude of 0.55 mm [16,21]. The procedure was as follows: prior to the vibration trials, each participant performed a warm-up for 10 min (5 min running with 5 min static stretching) and subsequently received 30 s vibration stimulation under each condition. After that, the RPE was measured immediately. The time interval between the conditions was 2 min. 

Three accelerometers with the Dactron Photon II spectrum analyzer (PCB Piezotronics Inc., Depew, NY, USA) were attached on the vibration platform, the left knee, and the head of the participants. According to the accelerometer placement of previous studies [18,27], the accelerometer (model: 353B34, weight: 27 g, sensitivity: 100 mV/g, amplitude range: ±50 g) of the vibration platform was fixed on the top surface and at the center. Moreover, the knee and head accelerometers (model: Y352C22/030C10, weight: 0.5 g, sensitivity: 10 mV/g, amplitude range: ±500 g) were fixed, respectively, at lateral epicondyle of the femur and at a bite bar, which was clenched between the opposing molar teeth. Each accelerometer was aligned with the same orientation and the acceleration of the axis was aligned to the direction of gravity and then recorded for data analysis. The sampling rate was set at 1000 Hz. During WBV exposure, the acceleration data obtained for analysis were from the 10th to 20th seconds when the participants reached a stable phase. The raw data collected were first smoothed using a Butterworth low-pass filter with a cutoff frequency of 80 Hz. The root-mean-square values of the acceleration for a 10 s window were calculated to quantify the vibration magnitude at the head, knee, and vibration platform during each WBV trial. The unit was represented as “g” (Earth’s gravitational acceleration). The vibration transmission was computed as the ratio of root-mean-square acceleration of the head or knee to that of the vibration platform and represented as the transmissibility of each segment. A transmissibility above 1.0 represented that the vibration acceleration transmitted to the segment was magnified, while a transmissibility below 1.0 represented that the vibration acceleration transmitted to the segment was mitigated from the platform.

Furthermore, after completing each condition, the participants were asked of their perceived magnitude of vibration immediately on overall discomfort, and the results were recorded according to the Borg CR10 Scale from 0 (“nothing at all”) to 10 (“extremely strong/almost maximal”) [35].

All values were represented by mean and standard deviations. SPSS 19 software (IBM, Armonk, NY, USA) was used for statistical analysis. One-way repeated measures ANOVA was conducted for comparisons of each variable among different angles. When significant differences were observed, Bonferroni corrections were applied for pairwise comparisons. Pearson’s correlation was used to probe the correlation between the magnitudes of RPE and the platform-to-head or platform-to-knee transmissibilities. The correlations were performed separately in different angles, since the knee angles were a confounding factor. The significance level was set at *p* ≤ 0.05. The effect sizes for all pairwise comparisons of each variable were calculated using Cohen’s d (d) and were computed using the G*Power analysis program (Version 3.1.9.2, Heinrich Heine Universität, Düsseldorf, Germany). Moreover, based on the scale for the magnitude of Cohen’s d difference, effect sizes of 0.20, 0.50, and 0.80 were respectively considered small, medium, and large [36].

## 3. Results

All of the participants completed each experimental condition without reporting any extreme discomfort or unusual symptoms during the experimental period. The accelerations of the platform ranged from 0.26 to 0.28 g and no significant difference of the acceleration among conditions was observed (*p* = 0.39), which indicated that the vibration intensity of each condition was the same.

**Figure 2:**
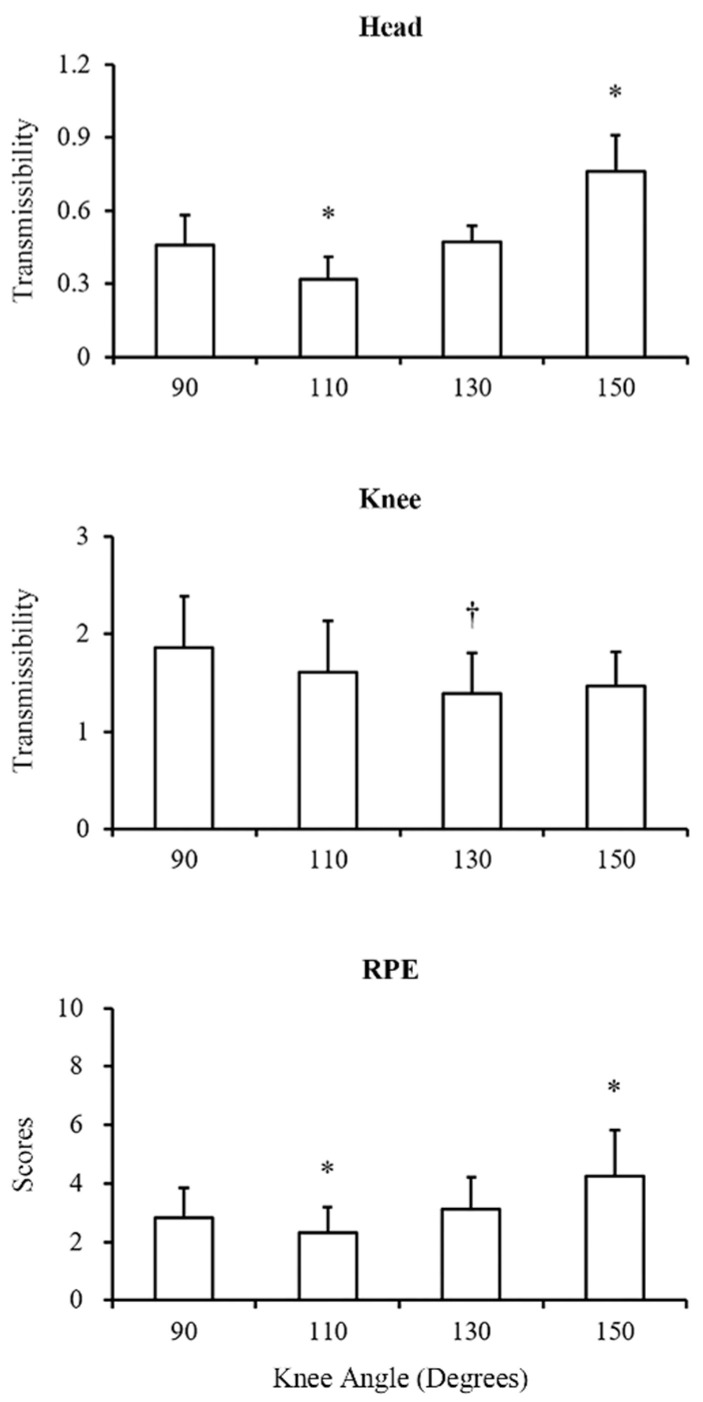
Comparison of the head transmissibility, knee transmissibility, and rating of perceived exertion (RPE) among different knee angle conditions. Data are expressed as mean ± SD. * Represents significant difference with other conditions (*p* < 0.01). † Represents significant difference with the knee flexion angle of 90 degrees condition (*p* < 0.01).

After comparison of the platform-to-head transmissibility (Table 1 and Figure 2), the ANOVA test showed a significant difference among all conditions (*p* < 0.01). Further post-hoc analysis showed that when the knee joint angle was maintained at 110 degrees, the platform-to-head transmissibility was the lowest among all conditions (all *p* < 0.01 versus 110 degrees, d = 1.40 to 2.25). Moreover, when the knee joint angle was maintained at 150 degrees, the platform-to-head transmissibility was the greatest among all conditions (all *p* < 0.01 versus 150 degrees, d = 1.42 to 2.25). Regarding the comparison of the platform-to-knee transmissibility among all conditions (Table 1 and Figure 2), the ANOVA test showed a significant difference (*p* < 0.01). The post-hoc analysis reported that the knee flexed at 130 degrees induced smaller transmissibility than at 90 degrees (*p* < 0.01, d = 1.03). 

For the comparison of the RPE (Table 1 and Figure 2), the ANOVA test showed a significant difference among all conditions (*p* < 0.01). The post-hoc analysis reported that the knee flexed at 150 degrees had the significantly highest RPE (*p* = 0.02, *p* < 0.01, and *p* = 0.02 of the knee flexed at 90, 110, and 130 degrees versus 150 degrees, d = 0.86 to 1.21). The knee flexed at 110 degrees had the significantly lowest RPE (*p* = 0.01, *p* = 0.03, and *p* < 0.01 of the knee flexed at 90, 130, and 150 degrees versus 110 degrees, d = 0.92 to 1.21). 

The Pearson’s correlation results (Table 2) revealed a significantly positive correlation between the RPE and the platform-to-head transmissibility only under the condition of the knee flexed at 150 degrees (r = 0.508, *p* = 0.04), while no statistically significant correlation was observed between the RPE and the platform-to-knee transmissibility (*p* > 0.05). 

## 4. Discussion

The objective results of this study showed that squatting at certain knee angles produced distinct effects on the vibration transmission to the head and knee. The important finding of this study is that the knee flexed at 110 degrees induced lower transmissibility to the head than the other relatively high or low squat conditions. Changing the knee flexion angle has been reported as a critical role for the attenuation of vibration to protect the head region, and the two possible influential factors usually mentioned are muscle activation and muscle stiffness [27,30]. Increasing the knee flexion angle, leading to more muscle activation, could potentially damp the vibration transmission during WBV [24,27,28]. Thus, the knee flexion of 110 degrees could be a suitable posture to minimize the vibration impact, compared with that of 130 and 150 degrees. However, the knee flexed at 90 degrees did not induce lower transmissibility to the head than at 110 degrees in this study. The negated damping effect may result from increased muscle stiffness [29]. Despite additional muscle activation at the condition of 90 degrees, the net trade-off between muscle activation and muscle stiffness appears to reduce the damping capability of muscles when they are in a lengthened position. Certainly, such phenomena are necessary to investigate in further studies. 

In the study of Tankisheva et al. [27], no significant difference between the knee flexion of 110 and 135 degrees was observed in the vibration transmissibility to the head, but was in that to lumbar. In contrast, our results showed that a higher transmissibility to the head was found in the knee flexion of 110 degrees than 130 degrees. This difference may be due to different posture instructions of the upper body. The previous study instructed the participants to hold handles during WBV, leading to a higher transmissibility to the lumbar; however, the participants of our study were not holding anything. Despite holding a handle that could alter the vibration transmission to the upper body, the knee flexion of 110 degrees could mitigate the vibration impact on the upper body. 

Sports medicine research has found that WBV is a promising approach for the elderly [31] and individuals with knee injuries to enhance their strength and balance [1,3,4], and could be an alternative treatment for glucocorticoid-induced osteoporosis and osteoarthritis [10]. However, some studies have paid attention to vibration loading applied on knee joints. The accumulative vibration impact has the potential to impair musculoskeletal system [15,16], especially in individuals with knee-related injuries. The transmissibility to the knee has been paid less attention than that to the head because of inducing less perceived discomfort. Therefore, the appropriate training angle to protect the knee joint should be considered and addressed when designing a WBV protocol for long- and short-term training. The present study documented that a knee flexion of 90 degrees induced greater vibration transmissibility to the knee than that of 130 degrees. This result was the same as the outcome of the previous study [18]. Based on these results, WBV training with a knee flexion of 90 degrees should be noted for the elderly and individuals with knee-related injuries. Instead, a knee flexed at 130 degrees is suggested. 

RPE is a rapid method to evaluate the training intensity or perceived effort of each individual [35]. A high positive relationship has been found (r = 0.79) between RPE after WBV and muscle activation of the lower extremity during WBV, which can be used to qualify training loads and stimulation [32,33]. It has been found in past studies that the knee angle could be used to significantly modulate the head transmissibility during WBV exposure [22], which is in agreement with our finding. The factor of knee angle has a confounding effect on the association between the RPE and the head transmissibility. In our current study, correlations were conducted separately for different angles to specifically observe the association between head transmissibility and RPE. Under the condition of the knee flexed at 150 degrees (with a high mean transmissibility value [0.76]), the change of RPE was associated with the platform-to-head transmissibility (r = 0.508), while there were no significant correlations under the other conditions (with low mean transmissibility values [0.32–0.47]). The overall discomfort produced by the WBV protocol in different knee angles was relatively mild (mean RPE < 5). This means that measuring RPE after WBV exposure may be, at least partially, affected by the discomfort sensation of the head when the platform-to-head transmissibility reaches a certain level. Such a finding indicates an important application for training safety, though the correlation was moderate. When performing a training protocol that includes sets with the same intensity in long- and short-term WBV training, the transmissibility to the head may be increased by some factors (such as poor posture control and muscle fatigue). High-intensity WBV (2.5 g) causes unpleasant experiences in adults [14] and is considered unsafe if the magnitude of vibration transmitted to the head exceeds 1 g [37]. Excessive magnitudes of vibration stimulus to the head could increase susceptibility to motion sickness, vertigo, and visual impairment [11,12,13,14]. The magnitude of head vibration can be monitored by the change of vibration transmission to the head. The RPE after each training set may reflect the magnitude of head vibration transition during vibration exposure and is a clinical sign that can be used to evaluate the potential risk of head trauma. It is worthwhile to further investigate if RPE can be used as an alternative of conventional vibration measurement methods to evaluate the risk of developing head vibration-related symptoms.

There are several limitations in this study. First, this study only investigated the low-frequency and peak-to-peak amplitude of 20 Hz and 0.55 mm using a vertical WBV machine. The sample size of this study was relatively small, since the estimation of minimum sample size was 19 using the G*Power analysis program (with an alpha 0.05, effect size 0.80, and power 0.80), and the group consisted of young males only. Accordingly, further research using various vibration protocols, a larger sample size, and different groups is required to confirm and support our findings. Second, to be close to clinical practice, the knee joint angle only was controlled in this study, which may be influenced by other joints, such as hip joints. Third, this may not be sufficient for understanding the whole range between 90 and 150 degrees of knee flexion, although non-linear outcomes were shown among different knee angles (90, 110, 130, and 150 degrees). Thus, further studies in this regard are warranted to entirely fill the gap between 90 and 150 degrees. 

## 5. Conclusions

A knee flexed at 110 degrees was the optimal angle to minimize the vibration transmission to the head during WBV exposure. A knee flexion of 130 degrees induced lower transmissibility to the knee. There was a positive relationship between the vibration transmission to the head and the RPE when the head transmissibility reached a certain level. The present findings provide valuable insights and information of the appropriate posture (knee angle) and vibration-induced injury risks during WBV.

Considering the training safety, a knee flexion of 110 degrees is the appropriate selection for minimizing head impact during vibration exposure. A knee flexion of 130 degrees could be a suitable angle for minimizing knee impact. Apart from adopting static squat postures during WBV training, in view of a previous study [21], performing a dynamic squat is a strategy to avoid high vibration exposure of the same body segments. Accordingly, performing a dynamic squat with a range of knee flexion from 110 to 130 degrees could be used to reduce the vibration exposure of the same region. Measuring the RPE during WBV training could concurrently monitor dangers in the head region. Therefore, the risk of vibration-induced injuries can be managed not only by selecting the appropriate angle of knee flexion, but also by subjectively measuring the RPE.

## Figures and Tables

**Table 1:** Comparison of the head transmissibility, knee transmissibility, and rating of perceived exertion among knee angle conditions.

Knee Angle Condition	90 Degrees	110 Degrees	130 Degrees	150 Degrees	F Value
Head transmissibility	0.46 ± 0.12	0.32 ± 0.08	0.47 ± 0.07	0.76 ± 0.14	43.93 ^1^
Knee transmissibility	1.86 ± 0.51	1.61 ± 0.51	1.38 ± 0.41	1.47 ± 0.33	6.77 ^1^
Rating of perceived exertion	2.8 ± 1.0	2.3 ± 0.9	3.1 ± 1.1	4.3 ± 1.6	13.84 ^1^

Data are expressed as mean ± SD. ^1^ Represents significant difference among the four conditions (*p* < 0.01).

**Table 2:** Correlation (r) between the rating of perceived exertion (RPE) and the head or knee transmissibility. Correlation analyses were separately performed on data from different angles.

Transmissibility	RPE			
	90 Degrees	110 Degrees	130 Degrees	150 Degrees
Head	0.145	0.069	−0.136	0.508 ^1^
Knee	0.025	−0.129	0.040	0.127

Data are expressed as mean ± SD. ^1^ Represents a significant relationship (*p* < 0.05).

## Data Availability

The data presented in this study are available on request from the corresponding author. The data are not publicly available due to confidentiality agreements with participants.

## References

[1] Costantino C., Bertuletti S., Romiti D. (2018). Efficacy of whole-body vibration board training on strength in athletes after anterior cruciate ligament reconstruction: A randomized controlled study. Clin. J. Sport Med..

[2] Yang W.W., Chou L.W., Chen W.H., Shiang T.Y., Liu C. (2017). Dual-frequency whole body vibration enhances vertical jumping and change-of-direction ability in rugby players. J. Sport Health Sci..

[3] Pamukoff D.N., Montgomery M.M., Choe K.H., Moffit T.J., Vakula M.N. (2018). Effect of Whole-Body Vibration on Sagittal Plane Running Mechanics in Individuals With Anterior Cruciate Ligament Reconstruction: A Randomized Crossover Trial. Arch. Phys. Med. Rehabil..

[4] Zafar H., Alghadir A., Anwer S., Al-Eisa E. (2015). Therapeutic effects of whole-body vibration training in knee osteoarthritis: A systematic review and meta-analysis. Arch. Phys. Med. Rehabil..

[5] Luo J., McNamara B., Moran K. (2005). The use of vibration training to enhance muscle strength and power. Sports Med..

[6] Chen C.H., Liu C., Chuang L.R., Chung P.H., Shiang T.Y. (2014). Chronic effects of whole-body vibration on jumping performance and body balance using different frequencies and amplitudes with identical acceleration load. J. Sci. Med. Sport.

[7] Cardinale M., Bosco C. (2003). The use of vibration as an exercise intervention. Exerc. Sport Sci. Rev..

[8] Savage R., Billing D., Furnell A., Netto K., Aisbett B. (2016). Whole-body vibration and occupational physical performance: A review. Int. Arch. Occup. Environ. Health.

[9] Guglielmino C., Musumeci G. (2020). Early elbow osteoarthritis in competitive enduro motorcyclist. Scand. J. Med. Sci. Sports.

[10] Musumeci G., Loreto C., Leonardi R., Castorina S., Giunta S., Carnazza M.L., Trovato F.M., Pichler K., Weinberg A.M. (2013). The effects of physical activity on apoptosis and lubricin expression in articular cartilage in rats with glucocorticoid-induced osteoporosis. J. Bone Miner. Metab..

[11] Ishitake T., Ando H., Miyazaki Y., Matoba F. (1998). Changes of visual performance induced by exposure to whole-body vibration. Kurume Med. J..

[12] Yan J.-G., Zhang L.-l., Agresti M., Yan Y., LoGiudice J., Sanger J.R., Matloub H.S., Pritchard K.A., Jaradeh S.S., Havlik R. (2015). Cumulative brain injury from motor vehicle-induced whole-body vibration and prevention by human Apolipoprotein AI molecule mimetic (4F) peptide (an Apo AI mimetic). J. Stroke Cerebrovasc. Dis..

[13] Amir I., Young E., Belloso A. (2010). Self-limiting benign paroxysmal positional vertigo following use of whole-body vibration training plate. J. Laryngol. Otol..

[14] Ljungberg J., Neely G., Lundström R. (2004). Cognitive performance and subjective experience during combined exposures to whole-body vibration and noise. Int. Arch. Occup. Environ. Health.

[15] Carlsöö S. (1982). The effect of vibration on the skeleton, joints and muscles: A review of the literature. Appl. Ergon..

[16] Kiiski J., Heinonen A., Järvinen T.L., Kannus P., Sievänen H. (2008). Transmission of vertical whole body vibration to the human body. J. Bone Miner. Res..

[17] International Organization for Standardization (ISO) (1997). Mechanical Vibration and Shock: Evaluation of Human Exposure to Whole-Body Vibration. Part 1: Mechanical Vibration and Shock. Evaluation of Human Exposure to Whole-Body Vibration. General Requirements.

[18] Muir J., Kiel D.P., Rubin C.T. (2013). Safety and severity of accelerations delivered from whole body vibration exercise devices to standing adults. J. Sci. Med. Sport.

[19] Perchthaler D., Horstmann T., Grau S. (2013). Variations in neuromuscular activity of thigh muscles during whole-body vibration in consideration of different biomechanical variables. J. Sports Sci. Med..

[20] Ritzmann R., Gollhofer A., Kramer A. (2013). The influence of vibration type, frequency, body position and additional load on the neuromuscular activity during whole body vibration. Eur. J. Appl. Physiol..

[21] Munera M., Bertucci W., Duc S., Chiementin X. (2016). Transmission of whole body vibration to the lower body in static and dynamic half-squat exercises. Sports Biomech..

[22] Lienhard K., Vienneau J., Friesenbichler B., Nigg S., Meste O., Nigg B.M., Colson S.S. (2015). The effect of whole-body vibration on muscle activity in active and inactive subjects. Int. J. Sports Med..

[23] Avelar N.C., Ribeiro V.G., Mezencio B., Fonseca S.F., Tossige-Gomes R., da Costa S.J., Szmuchrowski L., Gripp F., Coimbra C.C., Lacerda A.C. (2013). Influence of the knee flexion on muscle activation and transmissibility during whole body vibration. J. Electromyogr. Kinesiol..

[24] Cochrane D.J., Loram I.D., Stannard S.R., Rittweger J. (2009). Changes in joint angle, muscle-tendon complex length, muscle contractile tissue displacement, and modulation of EMG activity during acute whole-body vibration. Muscle Nerve.

[25] Nawayseh N. (2019). Transmission of vibration from a vibrating plate to the head of standing people. Sports Biomech..

[26] Caryn R.C., Hazell T.J., Dickey J.P. (2014). Transmission of acceleration from a synchronous vibration exercise platform to the head. Int. J. Sports Med..

[27] Tankisheva E., Jonkers I., Boonen S., Delecluse C., van Lenthe G.H., Druyts H.L., Spaepen P., Verschueren S.M. (2013). Transmission of whole-body vibration and its effect on muscle activation. J. Strength Cond. Res..

[28] Wakeling J.M., Nigg B.M., Rozitis A.I. (2002). Muscle activity damps the soft tissue resonance that occurs in response to pulsed and continuous vibrations. J. Appl. Physiol..

[29] Lin D.C., Rymer W.Z. (1998). Damping in reflexively active and areflexive lengthening muscle evaluated with inertial loads. J. Neurophysiol..

[30] Burke D., Hagbarth K.E., Löfstedt L., Wallin B.G. (1976). The responses of human muscle spindle endings to vibration of non-contracting muscles. J. Physiol..

[31] Cristi C., Collado P.S., Marquez S., Garatachea N., Cuevas M.J. (2014). Whole-body vibration training increases physical fitness measures without alteration of inflammatory markers in older adults. Eur. J. Sport Sci..

[32] Marin P.J., Santos-Lozano A., Santin-Medeiros F., Delecluse C., Garatachea N. (2011). A comparison of training intensity between whole-body vibration and conventional squat exercise. J. Electromyogr. Kinesiol..

[33] Bertucci W., Arfaoui A., Duc S., Letellier T., Brikci A. (2015). Effect of whole body vibration in energy expenditure and perceived exertion during intense squat exercise. Acta Bioeng. Biomech..

[34] Paddan G., Griffin M. (1988). The transmission of translational seat vibration to the head--I. vertical seat vibration. J. Biomech..

[35] Borg G. (1998). Borg’s Perceived Exertion and Pain Scales.

[36] Cohen J. (1988). Statistical Power Analysis for the Behavioral Sciences.

[37] Sonza A., Volkel N., Zaro M.A., Achaval M., Hennig E.M. (2015). A whole body vibration perception map and associated acceleration loads at the lower leg, hip and head. Med. Eng. Phys..

